# Transcription factors Krüppel-like factor 4 and paired box 5 regulate the expression of the Grainyhead-like genes

**DOI:** 10.1371/journal.pone.0257977

**Published:** 2021-09-27

**Authors:** Grzegorz Kotarba, Agnieszka Taracha-Wisniewska, Michal Miller, Michal Dabrowski, Tomasz Wilanowski

**Affiliations:** 1 Faculty of Biology, Institute of Genetics and Biotechnology, University of Warsaw, Warsaw, Poland; 2 Laboratory of Bioinformatics, Nencki Institute of Experimental Biology of Polish Academy of Sciences, Warsaw, Poland; Wake Forest School of Medicine: Wake Forest University School of Medicine, UNITED STATES

## Abstract

Genes from the Grainyhead-like (*GRHL*) family code for transcription factors necessary for the development and maintenance of various epithelia. These genes are also very important in the development of many types of cancer. However, little is known about the regulation of expression of *GRHL* genes. Previously, there were no systematic analyses of the promoters of *GRHL* genes or transcription factors that bind to these promoters. Here we report that the Krüppel-like factor 4 (KLF4) and the paired box 5 factor (PAX5) bind to the regulatory regions of the *GRHL* genes and regulate their expression. Ectopic expression of KLF4 or PAX5 alters the expression of *GRHL* genes. In KLF4-overexpressing HEK293 cells, the expression of *GRHL1* and *GRHL3* genes was upregulated by 32% and 60%, respectively, whereas the mRNA level of *GRHL2* gene was lowered by 28% when compared to the respective controls. The levels of *GRHL1* and *GRHL3* expression were decreased by 30% or 33% in PAX5-overexpressing HEK293 cells. The presence of minor frequency allele of single nucleotide polymorphism rs115898376 in the promoter of the *GRHL1* gene affected the binding of KLF4 to this site. The evidence presented here suggests an important role of KLF4 and PAX5 in the regulation of expression of *GRHL1-3* genes.

## Introduction

The Grainyhead-like (*GRHL*) genes belong to the *TFCP2*/Grainyhead family, and can be found in all animal species and fungi that were studied so far [1]. In mammals this family has two distinct branches, the Grainyhead-like subfamily termed *GRHL1-3* [2,3] and the other subfamily consisting of three genes: *TFCP2*, *TFCP2L1* and *UBP1*, which code for transcription factors with the same names (recently reviewed in [4,5]). The expression of the GRHL factors is tissue- and developmentally-specific, and they are found primarily in epithelial tissues, in organs such as epidermis, oral and olfactory epithelium, kidneys and urogenital tract, stomach and the digestive tract, heart and lung [6]. These factors are involved in a wide variety of biological processes including: skin barrier formation [7,8], neural tube closure [9,10], wound healing [8,11] and cancer development [12–14]. Furthermore, they are not merely passive markers of tumor growth, but they also directly influence the process of carcinogenesis [12–14]. Although this gene family plays a pivotal role in epithelial maintenance and development, no systematic analyses have ever been carried out to identify and characterize transcription factors binding to promoters or enhancers of *GRHL1-3* genes. The aim of our research was to fill this gap in our knowledge. The results from our previous works as well as literature analyses allowed us to propose two transcription factors that could potentially regulate the expression of *GRHL1-3* genes: the Krüppel-like factor 4 (KLF4) and the paired box 5 factor (PAX5). KLF4 is highly expressed in the epidermis where it is responsible for the formation of skin barrier [15]. The deficiency of this transcription factor is correlated with increased cell proliferation and skin tumorigenesis [16,17]. Previously we observed that in mice lacking the functional *Grhl3* gene the hyperproliferative defect in their epidermis is similar to another barrier defective mouse model, the *Klf4*-nullizygous mice, indicating that loss of these transcription factors leads to analogous phenotypic aberrations [18,19]. In addition, both GRHL3 and KLF4 are involved in wound healing [8,11,20]. The results from our more recent studies indicate that some single nucleotide polymorphisms (SNP), located in the promoter regions of genes from the *GRHL* family, occur with statistically significantly altered frequencies in patients with clear cell renal cell carcinoma (ccRCC) and non-melanoma skin cancer (NMSC) [21,22]. Our bioinformatics analyses revealed that the presence of one of these polymorphisms in patients with ccRCC may alter the binding of KLF4 [22]. PAX genes are critically required during embryogenesis and play important roles in cancer progression. PAX5 was found to interact with the promoter of human telomerase reverse transcriptase (*hTERT*) gene and regulate its transcription, and GRHL2 also regulates the expression of *hTERT* [23,24]. More links between PAX5 and *GRHL* genes were discovered in our studies of the midbrain-hindbrain boundary development in the zebrafish model, showing that the formation of this boundary is governed by the interplay between multiple transcription factors including PAX2/5/8 and GRHL2 orthologue–GRHL2B [25,26]. On the basis of these observations we put forward a hypothesis that KLF4 and PAX5 may regulate the expression of genes from the *GRHL* family. Moreover, the presence of different alleles of SNPs that affect transcription factor binding sites in the promoter regions of *GRHL* genes, may result in altered expression of these genes. For all of the above reasons, we carried out bioinformatics analyses of the regulatory regions for each gene: *GRHL1*, *GRHL2* and *GRHL3*, and identified potential KLF4 and PAX5 binding sites. Subsequently we carried out experiments to investigate whether these transcription factors bind to the regulatory regions of *GRHL* genes and regulate their expression. We also examined whether the presence of minor frequency allele of SNP rs115898376 in the promoter of *GRHL1* gene alters the binding of KLF4 and affects the level of expression of *GRHL1*. Our results confirmed that KLF4 and PAX5 regulate the expression of *GRHL* genes, and that SNP rs115898376 in the promoter of *GRHL1* gene affects the binding of KLF4 to this binding site.

## Materials and methods

### *In silico* prediction of KLF4 and PAX5 binding sites in the regulatory elements of *GRHL* genes

**Fig 1:**
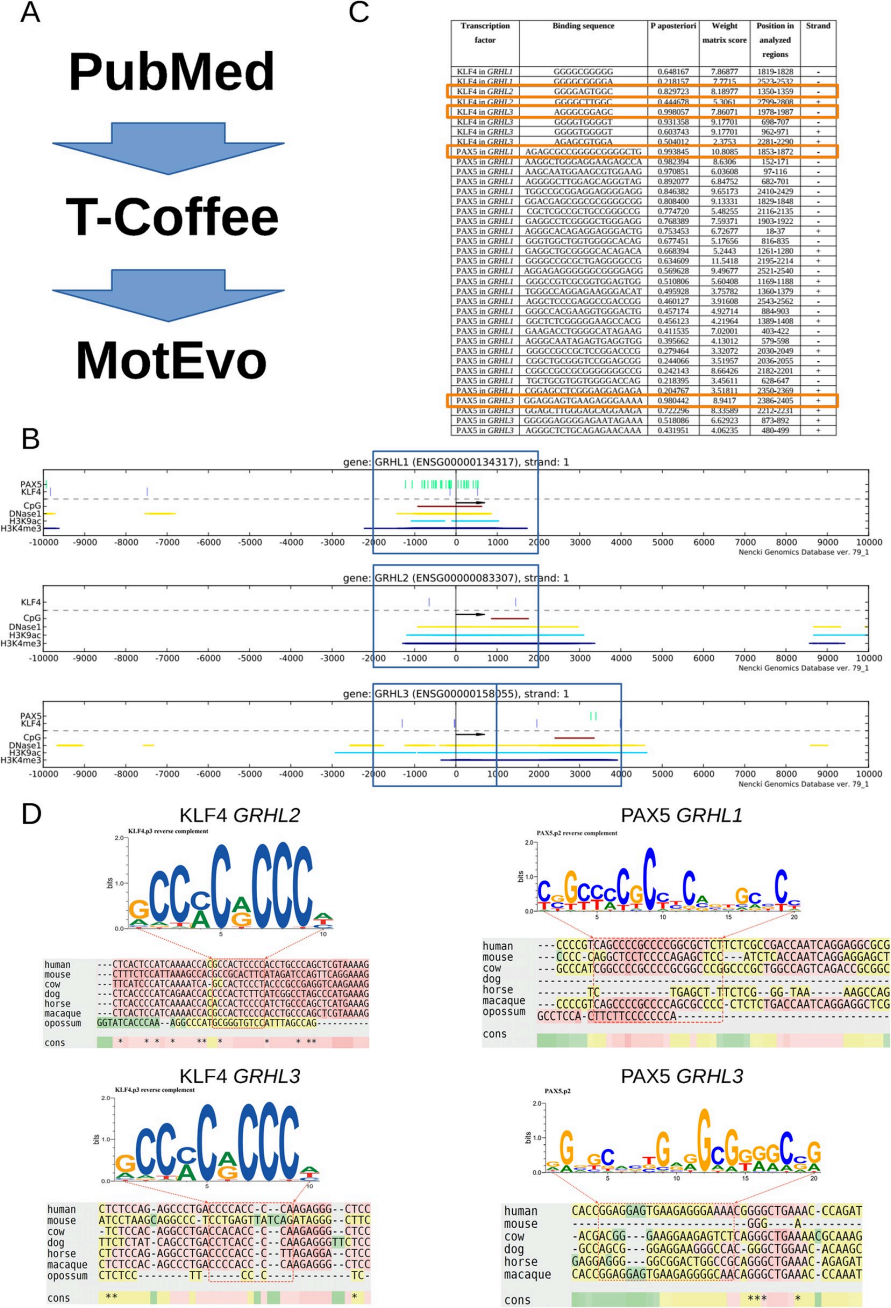
Bioinformatics predictions of TFBS motifs for KLF4 and PAX5 in the promoters of human *GRHL1-3* genes. (**A**) The overall analysis workflow, starting from the selection of the two transcription factors of interest that was based on the literature (PubMed). (**B**) Analysis windows used for multi-alignment, based on promoter features (DNase I-seq, CpG, H3K9ac, H3K4me3) from Ensembl v.79 are marked as blue rectangles within the ± 10 kb flanks of the TSS. Instances of SwissRegulon TFBS motifs for KLF4 and PAX5 in the promoter taken from the NGD database [27] are also shown. The plots were generated using the webservice of the NGD database [27], accessed with the client PlotGenomic.t2flow, which is provided as S1 File. In this file, all the analysis parameters are provided as example values, viewable and usable upon opening the client in Taverna Workbench [28] http://www.taverna.org.uk/download/workbench/. (**C**) Evolutionarily conserved TFBS motif instances for KLF4 and PAX5 identified using MotEvo [30] with the default parameters. The most conserved motif instances are marked by orange rectangles. (**D**) Multi-alignments around the most conserved instances of motifs KLF4 and PAX5.

Regulatory regions for further analyses were chosen based on Nencki Genomic Database (NGD) [27] which is convergent with Ensembl version 79. For *GRHL1* and *GRHL2* we selected 4000 bp regions flanking the transcription start sites (TSS) (2000 bp upstream and 2000 bp downstream of TSS). For *GRHL3* we selected a wider region of 6000 bp (2000 bp downstream of TSS and 4000 bp upstream), guided by its longer open chromatin and histone modification regions (Fig 1B). We analyzed the occurrence of transcription start sites, DNase I hypersensitivity sites, CpG islands and patterns of histone H3K9ac acetylation and H3K4me3 methylation (DNase I and histone modification data from any cell type) from Ensembl version 79 in the promoters of *GRHL* genes and concluded that the above-listed regions are the most likely to contain promoters of these genes (Fig 1B). The data were visualized using the WSDL (Web Service Description Language) webservice of the NGD database, accessed with the client PlotGenomic.t2flow provided as S1 File. In this file, all the analysis parameters mentioned above are explicitly provided as example values, viewable and usable upon opening the client in Taverna Workbench [28] http://www.taverna.org.uk/download/workbench/. The genomic coordinates of the selected TSS flanks of the *GRHL1-3* genes are provided in the “Analyzed regions” table in S2 File. For each of these human regions, we downloaded (from Ensembl ver. 79) its pairwise genomic alignments with orthologous sequences from 6 mammalian species: rhesus macaque (rheMac), mouse (mm), dog (canFam), horse (equCab), cow (bosTau), and opossum (monDom). This was achieved via the Ensembl Genome Browser (ver. 79), using the options: Configure this page, Comparative genomics, BLASTz/LASTz alignments; after choosing the orthologous species and the genomic coordinates of the human region; and the output was saved in the Fasta format. Multiple alignments were performed with T-Coffee [29]. Prior to using the pairwise alignments as the input for T-Coffee all the gaps in them were manually removed. We used a command line Version_12.00.7fb08c2 of T-Coffee, run with this command:./t_coffee -in = my_fasta.txt -mode = regular -output = score_html clustalw_aln fasta_aln score_ascii phylip -maxnseq = 150 -maxlen = 60000 -case = upper -seqnos = off -outorder = input -run_name = result -multi_core = 4 -quiet = stdout. For predicting transcription factor binding sites (TFBS) we used MotEvo [30] (ver 1.0) with standard settings, including position weight matrix (PWM) and phylogenetic tree ((((hg:0.048,rheMac:0.048): 0.143,mm: 0.489): 0.030((canFam: 0.224, equCab: 0.149): 0.011,bosTau:0.246):0.047):0.365,monDom:0.481). This phylogenetic tree was used by the authors of MotEvo in their original publication [30]. We used TFBS motifs for KLF4 (KLF4.p3) and PAX5 (PAX5.p2) from the Swiss Regulon [31] motif library for maximum compatibility with MotEvo [30] developed by the same authors. In analyses of regions located downstream and upstream of TSS we considered only those TFBSs which met the requirements of threshold that we set (p aposteriori > 0.8 and weight matrix (WM)-score > 8). Additionally, for KLF4, we chose the previously identified KLF4 motif site within the SNP rs115898376 (C/T) and also weaker motifs (p aposteriori < 0.8 and WM-score < 8) [22]. For the comparison of the MotEvo predictions with the ChIP-seq data from the Cistrome database [32] we used the function “What factors bind in your interval?” of http://dbtoolkit.cistrome.org/.

### Cell culture

Human embryonic kidney 293 cells (HEK293) were a kind gift from Ewelina Szymanska. The cell line was authenticated by LGC Standards (Teddington, Middlesex, UK). The cells were maintained in DMEM GlutaMAX medium supplemented with 10% fetal bovine serum (FBS) and 100 IU/mL penicillin-streptomycin at 37°C in a humidified atmosphere with 5% CO_2_. Cell culture components were purchased from Thermo Fisher Scientific.

### Plasmids

The pcDNA3.1-HA-KLF4 FL plasmid was a kind gift from Michael Ruppert (Addgene plasmid #34593) [33], pcDNA3.1-N-DYK-PAX5 plasmid was purchased from GenScript, the control pcDNA3.1 plasmid was purchased from Invitrogen. In co-transfection experiment no. 1, using In-Fusion^®^ HD Cloning Kit (Takara Bio), according to the protocol provided by the manufacturer, synthetic oligonucleotides of regulatory elements of *GRHL1* (50 bp from -429 to -379 with respect to the transcription start site, with KLF4 binding site or 40 bp identical fragment without KLF4 binding site), *GRHL2* (60 bp from -672 to -612 or 50 bp identical fragment without KLF4 binding site) and *GRHL3* (60 bp from -1324 to -1264 or 50 bp identical fragment without KLF4 binding site) or synthetic oligonucleotides of regulatory elements of *GRHL1* (60 bp from -180 to -120 or 40 bp identical fragment without PAX5 binding site) and *GRHL3* (60 bp from +3360 to +3420 or 40 bp identical fragment without PAX5 binding site) were cloned into the firefly luciferase vector with SV40 promoter (pGL3-promoter) (Promega). These plasmids were termed KLF4/GRHL1-luc, NoKLF4/GRHL1-luc, KLF4/GRHL2-luc, NoKLF4/GRHL2-luc, KLF4/GRHL3-luc, NoKLF4/GRHL3-luc, PAX5/GRHL1-luc, NoPAX5/GRHL1-luc, PAX5/GRHL3-luc, NoPAX5/GRHL3-luc, respectively. In co-transfection experiment no. 2, using the above mentioned In-Fusion^®^ HD Cloning Kit (Takara Bio) the *GRHL1* promoter fragment (synthetic oligonucleotides 50 bp fragment from -429 to -379 with major or minor frequency allele of SNP rs115898376), were cloned into the firefly luciferase vector with SV40 promoter (pGL3-promoter) (Promega). These plasmids were termed KLF4/GRHL1-luc2 and KLF4/GRHL1-SNP-luc2. All constructs were verified by sequencing. The control vector with *Renilla* luciferase gene (pRL-CMV) was also purchased from Promega. Fragments and primers used for cloning are listed in S1 Table.

### Chromatin Immunoprecipitation assays (ChIP)

HEK293 cells cultured in 6-well plates (well diameter 34.8 mm) were transfected with 1.0 μg of pcDNA3.1-HA-KLF4 FL or pcDNA3.1-N-DYK-PAX5 plasmid, using Lipofectamine 3000 (Thermo Fischer Scientific) according to the protocol provided by the manufacturer. After 24 h cells were crosslinked with 1% formaldehyde for 10 min, and subjected to sonication (25 min, 30 s on/30 s off) (Bioruptor, Diagenode) to generate ~500 bp DNA fragments. Chromatin was isolated with the Imprint Chromatin Immunoprecipitation Kit (Sigma-Aldrich), according to the manufacturer’s instructions. DNA fragments were immunoprecipitated with 3 μg of anti-KLF4 antibody (ab106629, Abcam) or 12.5 μg of anti-DYK (FLAG) antibody (ab1162, Abcam) and 1 μg of normal rabbit IgG as negative control (10500C, Thermo Fisher Scientific). The immunoprecipitated DNA was analyzed using quantitative real-time PCR (qPCR). To calculate real-time PCR results delta-delta cycle threshold (Ct) method was used. The fold changes related to 10% input delta Ct were calculated as 2^−ΔΔCt^ [34]. For negative control for KLF4 we used the *ZNF333* coding region (primers provided with the Imprint Chromatin Immunoprecipitation Kit). Negative control for PAX5 binding was *KRAS* gene which does not contain PAX5 binding sites [35]. Primers used for ChIP are listed in S2 Table. All ChIP assays were performed in triplicate. Statistical differences were determined by Student’s t-test. P ≤ 0.05 was considered statistically significant.

### Nuclear extract preparation and Electrophoretic Mobility Shift Assays (EMSA)

HEK293 cells grown on 100 mm plates were transfected (Lipofectamine 3000, Thermo Fischer Scientific) with 14.1 μg of pcDNA3.1-HA-KLF4 FL or pcDNA3.1-N-DYK-PAX5 plasmids. After 24 h, nuclear extracts were prepared using NE-PER Nuclear and Cytoplasmic Extraction Reagents (Thermo Fischer Scientific).

Binding reactions for EMSA were performed using 10 μg of nuclear extract proteins with 100 nM of 20–30 bp double-stranded 5’-fluorescein-labeled oligonucleotides (synthesized by Genomed) containing KLF4 or PAX5 binding site sequences with or without 100-fold molar excess of non-labeled double-stranded cold competitors in EMSA binding buffer: 10 mM Tris-HCl, pH 7.5; 50 mM KCl, 3.5 mM DTT, 0.25% Tween 20, 5 mM MgCl_2_, 50 ng/μl of poly(dI-dC) and 2.5% glycerol in a final volume of 20 μl. Binding reactions were incubated on ice for 30 minutes, after which protein-DNA complexes were separated by electrophoresis on 5% nondenaturing polyacrylamide 1 x Tris-borate-EDTA (TBE), 10% glycerol gel (120 V, 1.5 h) in 1 x TBE buffer and visualized on a Typhoon FLA 9000 laser scanner. To identify proteins bound to DNA probes, nuclear extracts were incubated with 3 μg anti-KLF4 antibody (ab106629, Abcam) or 1 μg of anti-PAX5 antibody (ab15164, Abcam) on ice, for 30 minutes prior to the addition of fluorescein-labeled DNA probes. The sequences of the oligonucleotides used in EMSA are listed in S3 Table.

### Total RNA extraction, reverse transcription and qRT-PCR assays

HEK293 cells cultured in 6-well plates were transfected with 1.0 μg of pcDNA3.1-HA-KLF4 FL or pcDNA3.1-N-DYK-PAX5, or pcDNA3.1-empty plasmids (negative control), using Lipofectamine 3000 (Thermo Fischer Scientific) in accordance with the manufacturer’s protocol. After 24 h, total RNA was extracted and purified using Total RNA Mini Plus Kit (A&A Biotechnology) following manufacturer’s instructions. The yield of RNA was estimated spectrophotometrically from absorbance at 260 nm, and RNA purity was evaluated according to the A_260_/A_280_ and A_260_/A_230_ ratio (NanoDrop ND-1000, Thermo Fisher Scientific). The integrity of total RNA was confirmed by the presence of sharp bands in UV light corresponding to 18S and 28S rRNA when separated by electrophoresis on 1.5% agarose. Next, 1 μg of total RNA was reverse-transcribed into first-strand cDNA with ReadyScript cDNA Synthesis Mix (Sigma-Aldrich). Quantitative real-time PCR was performer in triplicate using TaqMan Fast Universal PCR Master Mix No AmpErase UNG (Thermo Fischer Scientific) and TaqMan Probes (Thermo Fischer Scientific) on a StepOne Plus Real Time PCR system (Applied Biosystems). Gene expression levels were normalized to hypoxanthine phosphoribosyltransferase 1 housekeeping gene (*HPRT1*) (the highest stability based on literature [36]) and calculated using the 2^-ΔΔCt^ method [34]. The following TaqMan Probes were used in experiments: Hs01119372_m1 for *GRHL1*, Hs00227745_m1 for *GRHL2*, Hs00297962_m1 for *GRHL3*, Hs02800695_m1 for *HPRT1*. Statistical differences for relative expression levels were determined using Student’s t-test. P ≤ 0.05 was considered statistically significant.

### Reporter gene assays

HEK293 cells cultured in 6-well plates (well diameter 34.8 mm) were transfected (Lipofectamine 3000, Thermo Fischer Scientific) with 500 ng of pcDNA3.1-HA-KLF4 FL or pcDNA3.1-N-DYK-PAX5, or pcDNA3.1-empty plasmids, 25 ng pRL-CMV and 500 ng of the firefly luciferase vector with KLF4 or PAX5 binding sites (described in section *Plasmids*). Using the Dual-Luciferase Reporter Assay System (Promega), after 24 h, cells were lysed and luciferase activity was measured using Tecan Infinite M1000 PRO luminometer. Relative reporter activity was calculated and normalized based on *Renilla* luciferase activity. All experiments were carried out in triplicate. Statistical evaluations were performed using Student’s t-test. P ≤ 0.05 was considered statistically significant.

## Results

### Bioinformatics analyses of potential binding sites for KLF4 and PAX5 in the regulatory regions of the *GRHL1-3* genes

As detailed in the Introduction, the hypothesis that KLF4 and PAX5 may be biologically interesting transcription factors–regulators of expression of genes from the *GRHL* family was based on our previous work and analyses of scientific literature. Working with this hypothesis, we set ourselves a goal of bioinformatically identifying the most likely TFBSs for these two transcription factors in the vicinity of *GRHL1-3* gene promoters. The flow of our analysis is illustrated in Fig 1A. To identify the promoter regions of *GRHL* genes, we utilized transcription start sites established by the FANTOM consortium [37] and chromatin data (openness, histone methylation and acetylation patterns) from the ENCODE project [38], available via the NGD database [27]. From the NGD we also obtained the genomic coordinates of the instances of TFBS motifs for KLF4 and PAX5 (Fig 1B). In order to identify the most likely binding sites for either of these transcription factors, among the sometimes large numbers of instances of a particular TFBS motif in a given promoter, we employed phylogenetic footprinting with the MotEvo program [30], following multi-alignments of the regions orthologous to the analyzed promoter windows in 7 mammalian species with T-coffee [29]. MotEvo employs Bayesian reasoning and returns a posterior probability that a given TFBS motif’s instance is functional in the chosen species (here–human), given the motif strength in that species (Weight Matrix score) and its evolutionary conservation.

These bioinformatic analyses allowed us to identify 8 evolutionarily conserved putative binding sites for KLF4 in the regulatory regions of *GRHL1-3* genes and 29 evolutionarily conserved putative binding sites for PAX5 in the regulatory regions of *GRHL1* and *GRHL3* genes (Fig 1C and S4 Table). We did not find any evolutionarily conserved putative binding sites for PAX5 in the promoter region of *GRHL2*, and for this reason we excluded the PAX5/GRHL2 pair from further investigations. For experimental analyses for each *GRHL* gene we selected one predicted binding site for KLF4 or PAX5 (with the highest posterior probability (P aposteriori)), marked by orange rectangles in Fig 1C. The corresponding multi-alignments, matched to the logos of the respective TFBS motifs are shown in Fig 1D. We note that the selected predicted binding site for PAX5 in *GRHL3* is conserved in human and macaque only. All the potential binding sites for KLF4 and PAX5, together with their genomic coordinates and the human genome coordinates of the multi-aligned windows, are listed in S4 Table and S2 File. Moreover, for KLF4, we chose the previously identified KLF4 motif site with the SNP (C/T) (rs115898376) [22].

### KLF4 and PAX5 bind to the regulatory regions of the Grainyhead-like genes

**Fig 2:**
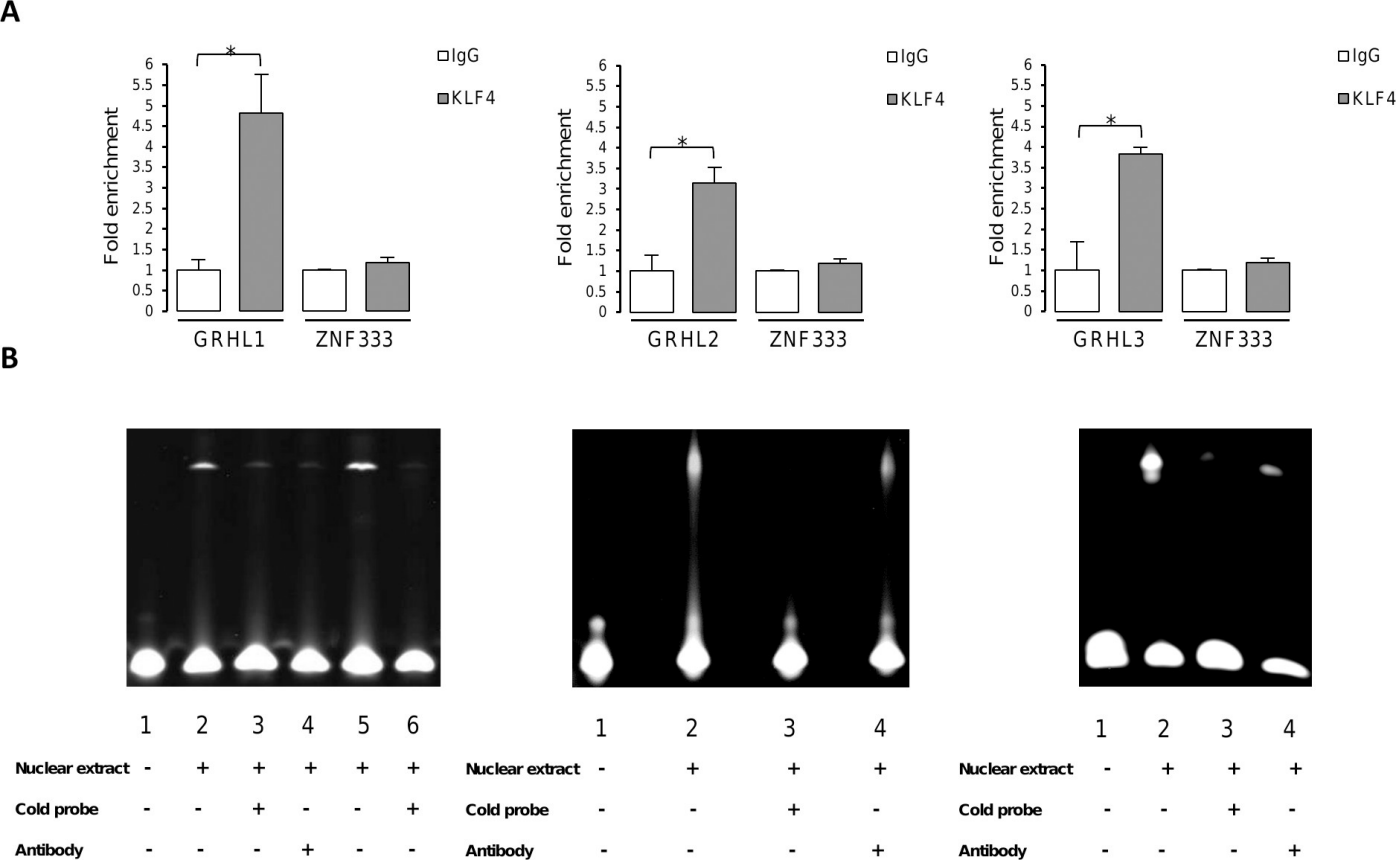
KLF4 binds to the regulatory regions of *GRHL* genes. (**A**) Quantitative ChIP-PCR analysis of KLF4 occupancy of *GRHL1*, *GRHL2* and *GRHL3* regulatory regions was performed in HEK293 cells transfected with pcDNA3.1-HA-KLF4 FL. *ZNF333* was used to identify non-specific interactions. Chromatin was immunoprecipitated with anti-KLF4 antibody or nonspecific antibody. The amount of DNA amplified from immunoprecipitated DNA was normalized to that amplified from input DNA. Data are shown as means ± SEM from experiments independently performed in triplicate, *significantly different at p≤ 0.05. (**B**) EMSA analysis performed with probes including KLF4 binding sequences: left panel–in the region -409/-400 (lane 2) or -409/-400 with the minor frequency allele of SNP rs115898376 (C/T) (lane 5) of the *GRHL1* promoter; central panel–in the region -650/-641 (lane 2) of the *GRHL2* promoter; right panel–in the region -1302/-1293 (lane 2) of the *GRHL3* promoter. Lane 1: probe only. Cold probe: unlabeled double-stranded oligonucleotides including -409/-400 (left panel, lane 3) or -409/-400 with SNP rs115898376 (C/T) (left panel, lane 6) or -650/-641 (central panel, lane 3) or -1302/-1293 (right panel, lane 3) regions of *GRHL* genes (100-fold molar excess of competitors). Where indicated, 3 μg anti-KLF4 antibody was added per lane (ab106629, Abcam).

**Fig 3:**
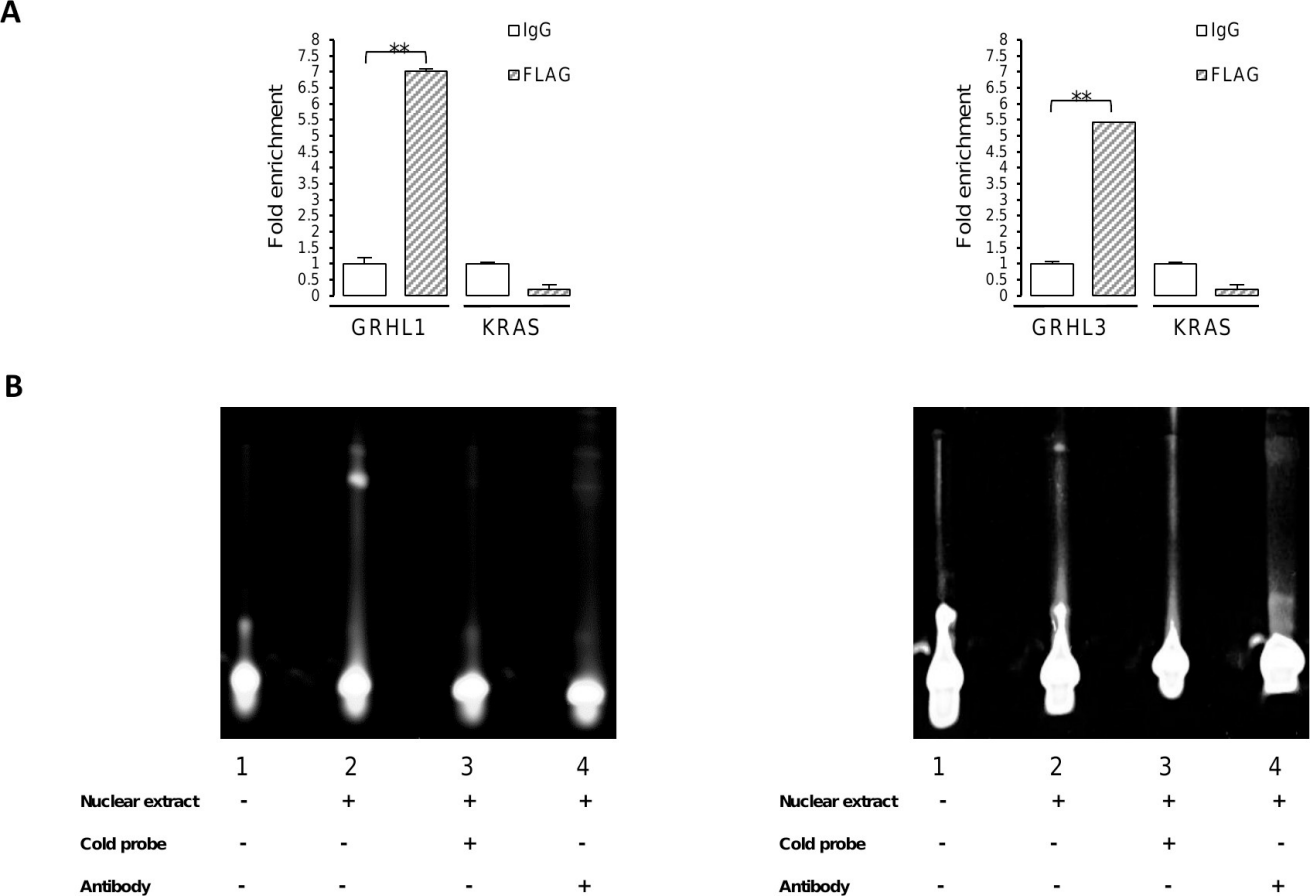
PAX5 binds to the regulatory regions of *GRHL* genes. (**A**) Quantitative ChIP-PCR analysis of PAX5 occupancy of the *GRHL1* and *GRHL3* regulatory regions was performed in HEK293 cells transfected with pcDNA3.1-N-DYK-PAX5. *KRAS* was used to identify non-specific interactions. Chromatin was immunoprecipitated with anti-DYK (FLAG) antibody or nonspecific antibody. The amount of DNA amplified from immunoprecipitated DNA was normalized to that amplified from input DNA. Data are shown as means ± SEM of experiments independently performed in triplicate, **significantly different at p≤ 0.01. (**B**) EMSA analysis performed with probes including PAX5 binding sequences: left panel–in the region -147/-128 (lane 2) of the *GRHL1* promoter; right panel–in the region +3386/+3405 (lane 2) of the *GRHL3* enhancer. Lane 1: probe only. Cold probe: unlabeled double-stranded oligonucleotides including -147/-128 (left panel, lane 3) or +3386/-3405 (right panel, lane 3) regions of *GRHL* genes (100-fold molar excess of competitors). Where indicated, 1 μg anti-PAX5 antibody was added per lane (ab15164, Abcam).

To determine whether the bioinformatically predicted binding sites in the regulatory regions of *GRHL* genes are direct targets for KLF4 and PAX5, ChIP analyses were carried out. Chromatin from HEK293 cells transfected with either KLF4 or DYK-tagged PAX5 expressing vectors was immunoprecipitated with anti-KLF4 or anti-FLAG antibody, or normal IgG, and then examined by qPCR using primers listed in S2 Table. As a result, we found that the investigated DNA fragments were significantly enriched by anti-KLF4 antibody in comparison with nonspecific IgG, indicating KLF4 binding to the sequences of *GRHL1*, *GRHL2* and *GRHL3* promoters (Fig 2A). The data also showed a strong enrichment of the qPCR signal with anti-FLAG antibody in comparison with normal IgG, demonstrating that PAX5 binds to the *GRHL1* promoter sequence and also to the enhancer sequence located in the first intron of *GRHL3* gene (Fig 3A). We found no enrichment of *ZNF333* or *KRAS* (negative controls for KLF4 or PAX5, respectively) qPCR signals, indicating that KLF4 and PAX5 binding was specific (Figs 2A and 3A).

Our ChIP results have shown that both KLF4 and PAX5 transcription factors bind to the predicted regulatory regions of *GRHL* genes. In order to confirm KLF4 and PAX5 binding site sequences (selected on the basis of bioinformatics analyses) in the *GRHL* regulatory elements, we designed a number of fluorescein-labeled oligonucleotide probes for EMSA experiments. We performed EMSA with nuclear extracts from HEK293 cells following their transfection with either KLF4 or DYK-tagged PAX5 expressing vectors. We discovered that KLF4 binds to oligonucleotide probes containing binding sites: -409/-400 of *GRHL1*, -650/-641 of *GRHL2* and -1302/-1293 of *GRHL3* promoters, as shown by the representative results in Fig 2B. Cold competitor probes containing KLF4 binding sites effectively competed with respective fluorescein-labeled probes. Interestingly, the -409/-400 fragment with the minor frequency allele of SNP rs115898376 (C/T) was also bound by KLF4. Anti-KLF4 antibody completely abolished the binding, indicating that the protein-DNA interactions were specific. Similarly, PAX5 interacted with labeled probes containing fragments -147/-128 of *GRHL1* promoter and +3386/+3405 of *GRHL3* enhancer. Results are shown in Fig 3B. A 100-fold molar excess of identical but unlabeled probes resulted in a decrease of fluorescence signal, and 1 μg of anti-PAX5 antibody eliminated protein-DNA complexes, showing that PAX5 binding was specific. Quantitative results of EMSA experiments are presented in S1 Fig.

### Ectopic expression of KLF4 or PAX5 alters the expression of *GRHL* genes

**Fig 4:**
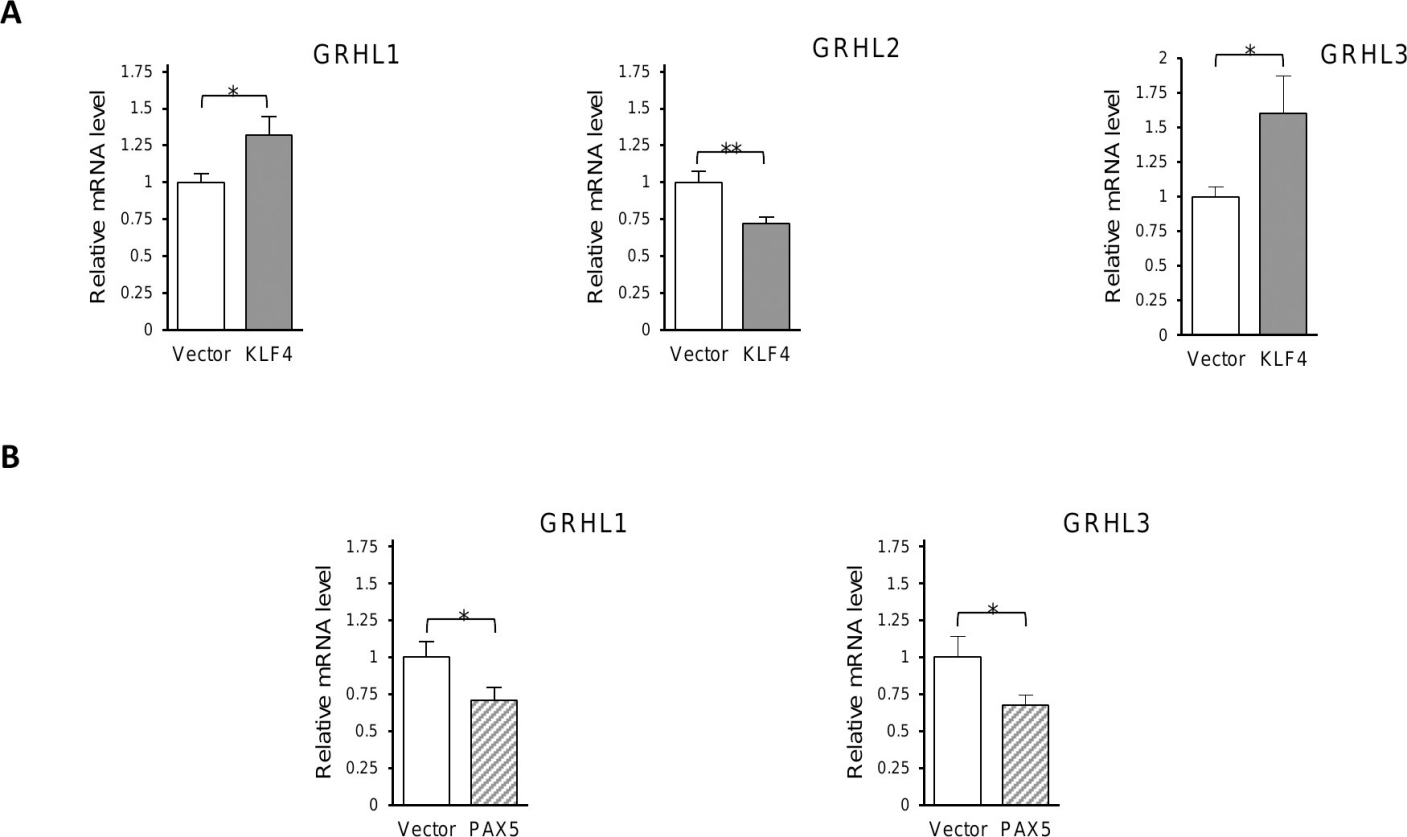
Overexpression of KLF4 or PAX5 alters mRNA levels of *GRHL* genes. (**A**-**B**) The mRNA expression levels of *GRHL1*, *GRHL2* and *GRHL3* genes in HEK293 cells transiently overexpressing KLF4 (**A**) or PAX5 (**B**). The results represent relative expression of the respective target gene vs *HPRT* gene. Data are shown as means ± SEM of experiments independently performed in triplicate, *significantly different at p≤ 0.05, **significantly different at p≤ 0.01.

By regulating the expression of its target genes, KLF4 may function as a suppressor or an oncogene in a context-dependent manner [39]. Contrary to its oncogenic effects in B cell lymphomas, PAX5 seems to play a role of an anti-proliferative protein in many malignancies [40–42]. However, it was still unknown whether these transcription factors regulate the expression of *GRHL* genes. For this purpose, HEK293 cells were transiently transfected with either KLF4 or PAX5 expressing plasmids, and the control empty vector. Next, using qRT-PCR we investigated the levels of expression of *GRHL1-3* genes. In KLF4-overexpressing HEK293 cells, the expression of *GRHL1* and *GRHL3* genes was upregulated by 32% or 60%, respectively, whereas the mRNA level of *GRHL2* gene was reduced by 28% when compared to the respective control cells (Fig 4A). We also observed a statistically significant decrease by 30% or 33% in the *GRHL1* and *GRHL3* mRNA levels in PAX5-overexpressing HEK293 cells (Fig 4B).

### KLF4 and PAX5 change the activity of *GRHL* regulatory regions

**Fig 5:**
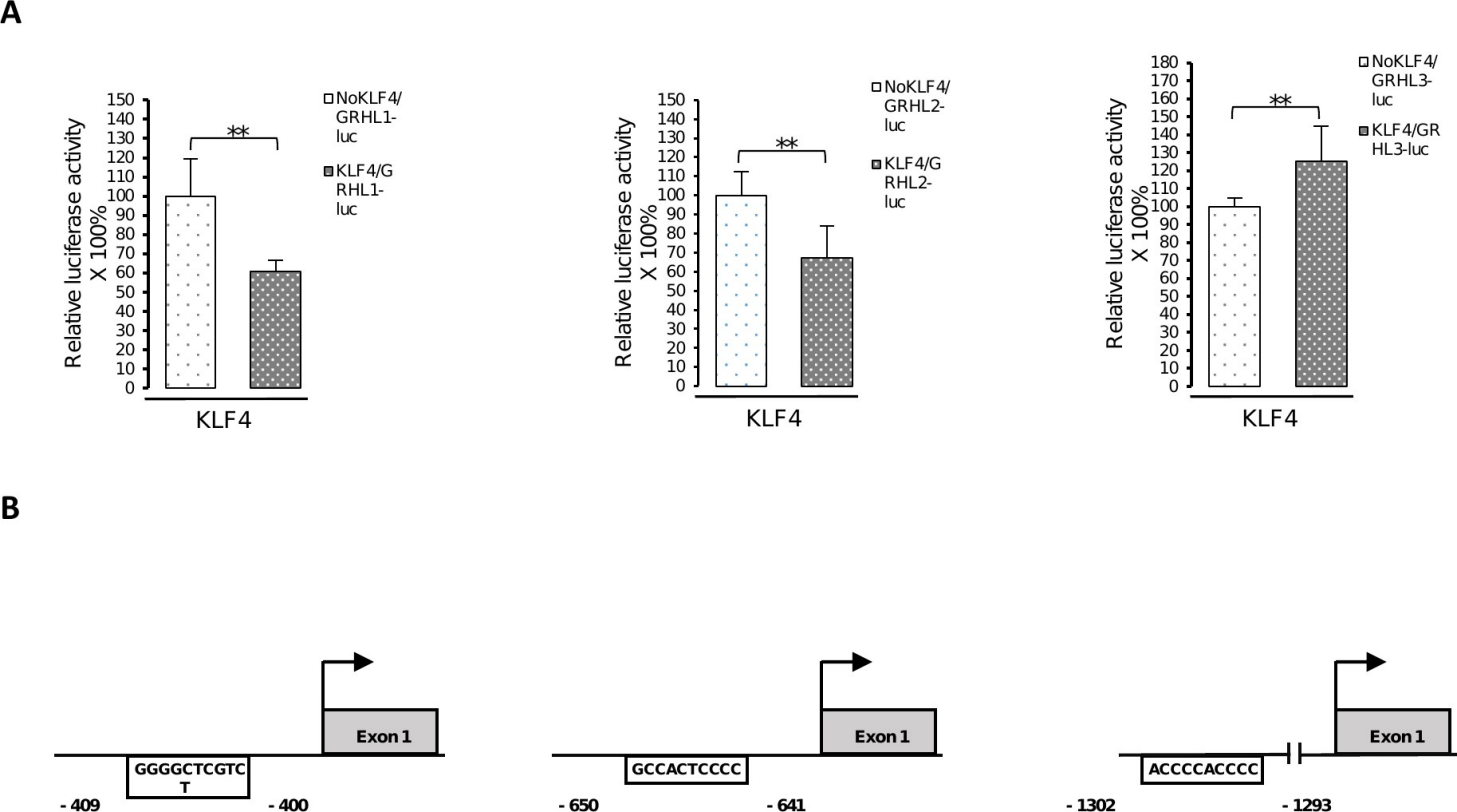
KLF4 changes the activity of *GRHL* regulatory regions. (**A**) HEK293 cells were transfected with pcDNA3.1-KLF4 plasmid, pGL3-promoter vector containing the luciferase gene under the control of the fragment of the *GRHL1* or *GRHL2* or *GRHL3* regulatory sequence with or without binding sites for KLF4 (named: KLF4/GRHL1-3-luc or NoKLF4/GRHL1-2-luc) and pRL-CMV *Renilla* luciferase control reporter vector. Data are shown as means ± SEM of experiments independently performed in triplicate, *significantly different at p≤ 0.05, **significantly different at p≤ 0.01. (**B**) Schematic representation of locations of the KLF4 binding site sequences in the promoters of *GRHL1*, *GRHL2* and *GRHL3* genes cloned into luciferase vectors.

**Fig 6:**
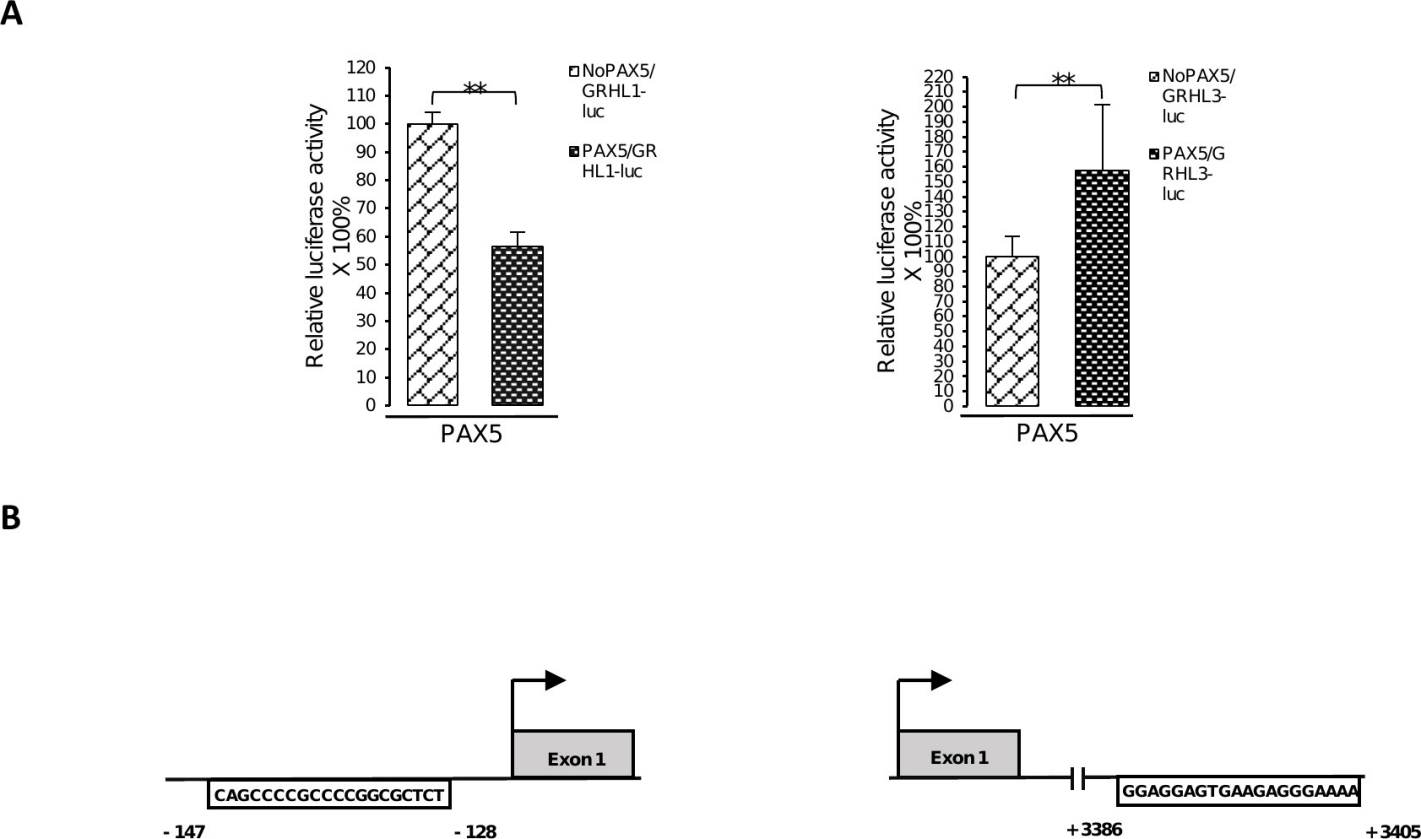
PAX5 changes the activity of *GRHL* regulatory regions. (**A**) HEK293 cells were transfected with pcDNA3.1-N-DYK-PAX5 plasmid, pGL3-promoter vector containing the luciferase gene under the control of the fragment of the *GRHL1* or *GRHL3* regulatory sequence with or without binding sites for PAX5 (named: PAX5/GRHL1-luc or PAX5/GRHL3-luc or NoPAX5/GRHL1-luc or NoPAX5/GRHL3-luc and pRL-CMV *Renilla* luciferase control reporter vector. Data are shown as means ± SEM of experiments independently performed in triplicate, **significantly different at p≤ 0.01. (**B**) Schematic representation of locations of the PAX5 binding site sequences in the promoter of *GRHL1* gene and in the enhancer of *GRHL3* gene cloned into luciferase vectors.

We performed co-transfection studies using fragments of regulatory regions of *GRHL* genes fused to the reporter gene–luciferase. In experiment no. 1 we transiently transfected HEK293 cells with reporter constructs containing *Luc* gene under the control of DNA fragments in which the KLF4 and PAX5 binding site sequences were either present or deleted. The results obtained indicate that in cells co-transfected with the vector including the KLF4 binding site, the expression of *Luc* gene fused to *GRHL1* promoter fragment was efficiently repressed (the *Luc* enzyme activity was downregulated by 39%). The *Luc* enzyme activity was also downregulated by 33% under the control of *GRHL2* promoter fragment and upregulated by 125% under the control of *GRHL3* promoter fragment (Fig 5A). Analogous experiment showed a decrease in luciferase activity from plasmid with *GRHL1* promoter (by 43%) and an increase in the case of a plasmid containing a *GRHL3* enhancer element (by 157%) in HEK293 cells co-transfected with the vector coding for PAX5 (Fig 6A).

**Fig 7:**
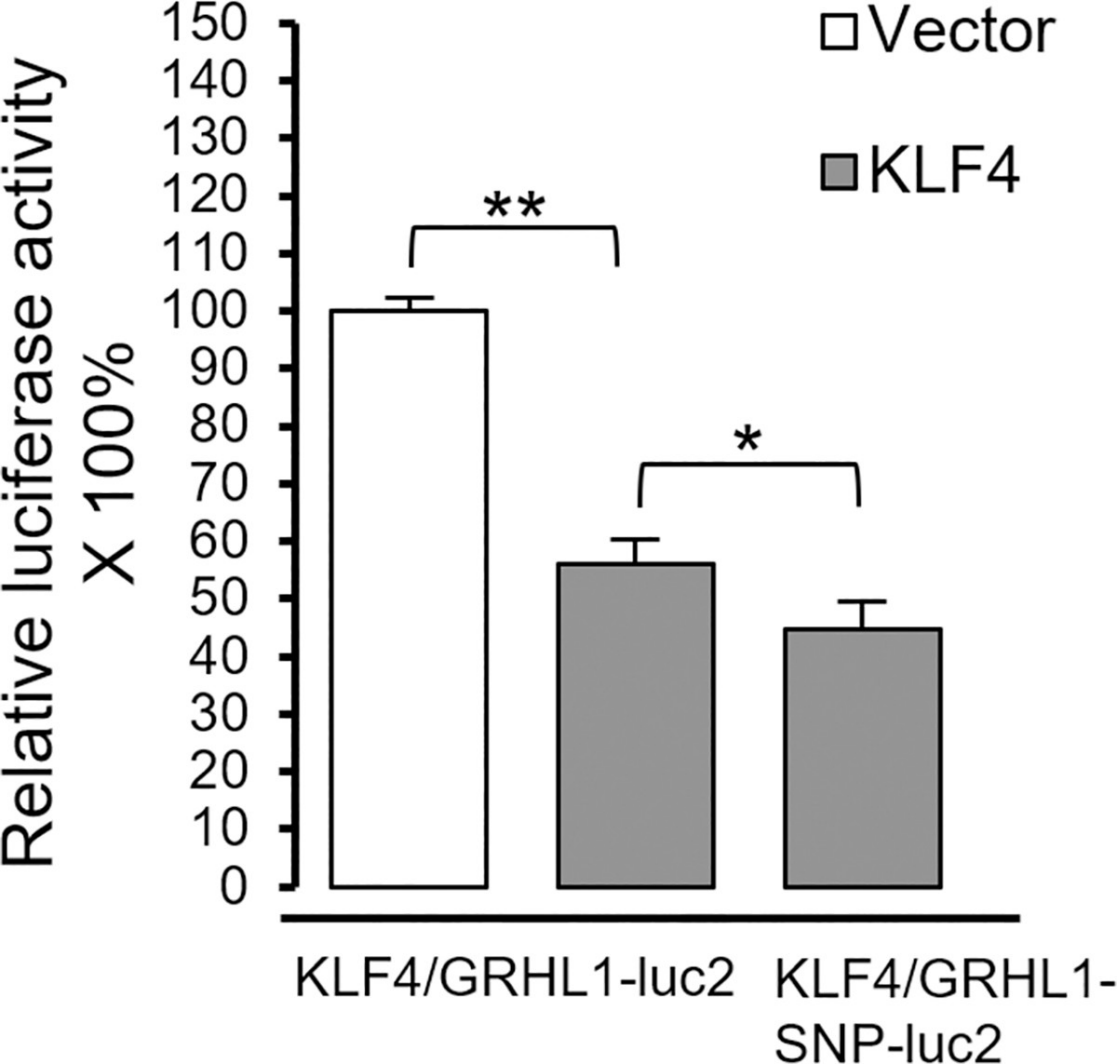
Impact of the presence of the minor frequency allele of SNP rs115898376 in the promoter of *GRHL1* gene on KLF4 binding. HEK293 cells were transfected with pcDNA3.1-KLF4 or pcDNA3.1-empty plasmid, pGL3-promoter vector containing the luciferase gene under the control of the fragment of the *GRHL1* regulatory sequence with either the major or the minor frequency allele of SNP rs115898376 (named KLF4/GRHL-luc2 and KLF4/GRHL1-SNP-luc2), and pRL-CMV *Renilla* luciferase control reporter vector. The presence of the minor frequency allele of SNP rs115898376 in the promoter of *GRHL1* gene alters the functioning of the KLF4 binding site.

In experiment no. 2 we transiently transfected HEK293 cells with reporter constructs with *Luc* gene under the control of DNA fragments containing KLF4 binding site sequences in the *GRHL1* promoter, with either the major frequency allele of SNP rs115898376 or the minor frequency allele of this SNP. Our results showed that, in cells co-transfected with the KLF4 expressing vector, the expression of *Luc* gene fused to *GRHL1* promoter fragment was efficiently repressed (the *Luc* enzyme activity was downregulated by 44%), moreover the presence of minor frequency allele of SNP rs115898376 in the cloned fragment of *GRHL1* gene resulted in further reduced expression (the *Luc* enzyme activity downregulated by further 20%) in comparison with the same region with the major frequency allele of this SNP (Fig 7).

## Discussion

The aim of our research was to identify additional transcription factors regulating the expression of genes from the *GRHL* family, because the precise regulation of the expression of *GRHL* genes is essential for their function and affects the functioning of encoded proteins. The role of GRHL transcription factors is well defined and is associated with the regulation of expression of genes involved in epithelial proliferation and differentiation [7,43]. Imbalance between these processes is frequently observed in a variety of diseases including atopic dermatitis, psoriasis, idiopathic pulmonary fibrosis and cancer [14,44,45]. To date, only fragmentary studies investigated the regulation of expression of *GRHL* genes by transcription factors. For this reason in the present work we employed comprehensive literature and bioinformatics analyses aimed at predicting which transcription factors may regulate *GRHL* expression. The completion of above analyses allowed us to identify KLF4 and PAX5 as putative regulators of *GRHL* expression. Our results show that KLF4 binds to the promoters of *GRHL1-3*, while PAX5 interacts with *GRHL1* and *GRHL3* regulatory regions. While most of these TF-binding results are novel, by comparison with the ChIP-seq data from the Cistrome database [32] we found out the PAX5 binding site that we focused on in *GRHL1* intersects a peak of PAX5-binding identified in the ChIP-seq experiment performed in lymphoblastoid B-cells (S2 File) [46]. We discovered that overexpression of KLF4 and PAX5 transcription factors in HEK293 cells have opposite effects on the mRNA levels of the *GRHL* genes. KLF4 positively regulates *GRHL1* and *GRHL3* expression and negatively regulates the *GRHL2* gene, while PAX5 has the ability to decrease *GRHL1* and *GRHL3* mRNA levels. Earlier studies revealed that the development of many types of cancer is often accompanied by changes in the levels of expression of the genes from the *GRHL* family [12–14]. Our previous work showed that the *GRHL1* and *GRHL3* expression is downregulated in the samples of human NMSC [21]. Also, our results indicated that the *Grhl1*-null mice display increased susceptibility to chemically-induced skin tumorigenesis [47] and deletion of *Grhl3* in the epidermis increases the occurrence of squamous cell carcinoma (SCC) of the skin [48]. Moreover, silencing of *GRHL2* expression in non-tumorigenic kidney cell line resulted in increased cell proliferation and increased resistance to apoptosis [22]. The above findings indicate that *GRHL1*, *GRHL2* and *GRHL3* serve protective role against NMSC and ccRCC. KLF4 plays a pivotal role in regulating various cellular processes including proliferation, differentiation, development, maintenance of homeostasis and apoptosis [49,50]. This transcription factor can function either as a tumor suppressor or as an oncogene depending on different cellular context [39]. Some studies showed that induction of KLF4 in basal keratinocytes initiates squamous epithelial dysplasia [51]. On the other hand, KLF4 is a tumor suppressor in a UVB-induced mouse skin tumor model [52] and its expression is decreased in samples from patients with SCC and basal cell carcinoma of the skin [53]. Additionally, knockdown of KLF4 in human epidermal squamous cell carcinoma SCC13 cell line was associated with increased cell growth, migration and adhesion [53]. Other studies revealed that KLF4 expression was lower in ccRCC tumors than in patient normal control samples both at the protein and mRNA levels, while the KLF4 overexpression arrested the cell cycle progress in ccRCC cell lines [54]. Given the above, KLF4 has an important function in tumorigenesis and it is possible that its role as a tumor suppressor in SCC and ccRCC is related to its ability to regulate the expression of all three *GRHL* genes. Furthermore, *Klf4*-null mice die immediately after birth due to the lack of an impermeable skin barrier [19], and the same phenotype is observed in the *Grhl3*-null mice [8]. Although all KLF factors recognize related sequences, KLF4 is the only KLF with an expression pattern specific to skin and gut [55]. The expression of KLF4 is unchanged in the *Grhl3*-null mice [8] which suggests that KLF4 is likely to regulate the expression of *Grhl3* in this context as well.

In this study we discovered that PAX5 negatively regulates the expression of *GRHL1* and *GRHL3*. PAX5 is expressed in aggressive N-type neuroblastoma cell lines, and downregulation of this transcription factor significantly reduced their proliferation rate and tumorigenic phenotype [56]. Moreover, PAX5 plays an important role during oral carcinogenesis where increase in PAX5 expression (at both mRNA and protein levels) was observed in oral SCC cell lines, compared to human normal oral keratinocytes [57]. We can assume that the oncogenic effects of PAX5 may be associated with its negative regulation of expression of *GRHL1* and *GRHL3*, since *GRHL1* has been shown to be a tumor suppressor in neuroblastoma [58], whereas *GRHL3* is a tumor suppressor in head and neck SCC [59].

In the present project we also attempted to investigate some of the functional binding sites for KLF4 and PAX5 in the regulatory regions of *GRHL* genes (Figs 5–7). Our results prove that all of the investigated DNA fragments respond to the regulation by KLF4 or PAX5, as predicted by us. All the changes in luciferase activity are statistically significant. However, the direction of these changes is not always consistent with the direction of changes of expression of endogenous *GRHL* genes upon overexpression of KLF4 or PAX5. The expression of *GRHL1* is increased upon ectopic overexpression of KLF4 (Fig 4A) but the reporter gene assays of one KLF4 binding site suggest that the opposite should be true (Figs 5A and 7). Similar discrepancy can be observed regarding the regulation of *GRHL3* by PAX5 (Figs 4B and 6A). These apparent contradictions can be explained by the findings that, at least in some cases, variation in the number of binding sites for a single transcription factor is sufficient to encode activation versus repression of the regulated gene [60]. In the promoter of the *GRHL1* gene we identified two putative binding sites for KLF4, while in the promoter of the *GRHL3* gene we identified four putative binding sites for PAX5 (Fig 1C and S4 Table). If simple rearrangements of TFBSs can encode qualitatively different responses to a single transcription factor [60] then it is not surprising that the effect of only one TFBS investigated in reporter assays can be opposite to the effect of an arrangement of a number of TFBSs in the endogenous locus. Another possible explanation for the above-mentioned discrepancies can be provided by “squelching” [61,62]. Overexpression of a transcriptional transactivator can sometimes repress the transcription of its target genes. This phenomenon is likely to be caused by the titration of limiting transcriptional coactivators [62]. “Squelching” in mammalian cells is limited to episomal target genes (such as our reporter gene constructs), but it does not affect the target genes present on cellular chromosomes [61], which is why the direction of expression changes shown in Fig 4 is likely to be correct. One more possible explanation is that the outcome of the transcription factor binding in the promoter of an endogenous gene is modified by interaction with enhancer(s), which are not reflected by the reporter construct. Nevertheless, our results prove that KLF4 and PAX5 bind to these sites, and that this binding has a regulatory effect.

We are cognizant of other shortcomings of overexpression experiments. Unbalancing gene dosage can lead to various artifacts and systemic errors, hence the results of overexpression experiments should be verified at native expression and/or *in vitro* [62]. To address this concern, we confirmed our findings by EMSA which is an *in vitro* assay based on a different principle (Figs 2 and 3). Our experiments were repeated only in triplicate, which is why our work is of preliminary nature. Nevertheless, our experimental results are consistent with the outcome of bioinformatic studies and literature analyses discussed above, which adds weight to the validity of our research. The observed changes in gene expression are relatively small (Figs 4–7). This can be explained by the fact that overexpression experiments are inherently quantitative and not qualitative [63]. Substantial fractions of overexpressed transcription factors can be present in inactive forms as they often require posttranslational modifications to fulfil their functions [63,64]. Future experiments will require animal models and samples obtained from human patients to determine whether the regulatory events studied by us are relevant in biology and medicine.

The results from our previous studies indicated that the minor frequency allele of SNP rs115898376, located in the promoter region of *GRHL1* gene, occurs at significantly altered frequencies in patients with ccRCC and the presence of this polymorphism may alter KLF4 binding [22]. In this work we confirmed this hypothesis to be true. We cloned 50 bp fragment of *GRHL1* promoter, with two putative KLF4 binding sites containing an either major or minor frequency allele of this SNP, into the plasmid with reporter luciferase gene. We found that co-transfection with KLF4 expressing plasmid decreased luciferase activity, moreover luciferase level was lower when using reporter vector containing the minor frequency allele of SNP indicating that the presence of this allele affected the binding of KLF4 to cloned fragment (Fig 7). However, the presence of the minor frequency allele of this SNP does not completely abolish KLF4 binding as in EMSA assays with probes containing major or minor frequency allele of SNP rs115898376 we found no difference in KLF4 binding ability (Fig 2B).

In conclusion, our results reveal that both KLF4 and PAX5, by binding to the regulatory regions of *GRHL* genes. are able to regulate the expression of these genes. Moreover, the presence of different alleles of SNP rs115898376 in the promoter region of the *GRHL1* gene alters KLF4 binding and influences its ability to regulate *GRHL1* transcription.

## Supporting information

S1 Fig Quantitative results of EMSA experiments performed with probes including KLF4 (**A**) or PAX5 (**B**) binding sequences. The relative decrease in signals for cold probe or probe with KLF4 or PAX5 antibody compared with probes with nuclear extract (100%) was measured by densitometry (ImageJ). Data are shown as means ± SEM of two or three experiments. * one experiment.
(PDF)

S1 Table List of oligonucleotides used for cloning. (DOC)

S2 Table List of ChIP qRT-PCR primers. (DOC)

S3 Table List of EMSA oligonucleotide probes. (DOC)

S4 Table List of identified putative binding sites for KLF4 and PAX5 in the regulatory regions of *GRHL1-3* genes. (DOC)

S1 File Taverna Workbench workflow (a NGD webservice client) used to prepare Fig 1B. (T2FLOW)

S2 File Genomic coordinates of binding sites for KLF4 and PAX5 in the promoter regions of *GRHL* genes. (XLS)

S1 Raw images (PDF)
