# Supplementary figures and images for: Transcription factors Krüppel-like factor 4 and paired box 5 regulate the expression of the Grainyhead-like genes
Source: PLoS One. 2021 Sep 27;16(9):e0257977. doi: 10.1371/journal.pone.0257977 (PMC8476022; doi:10.1371/journal.pone.0257977)

**A**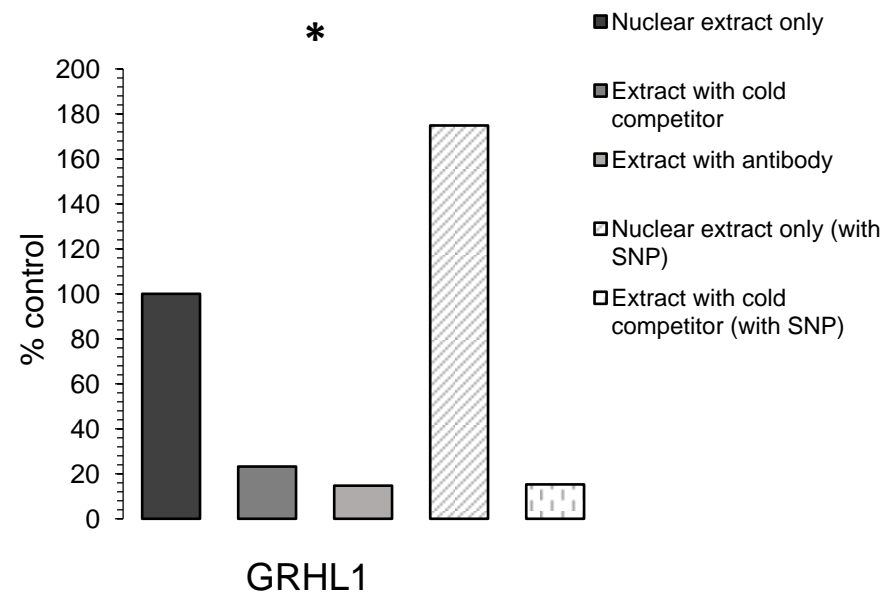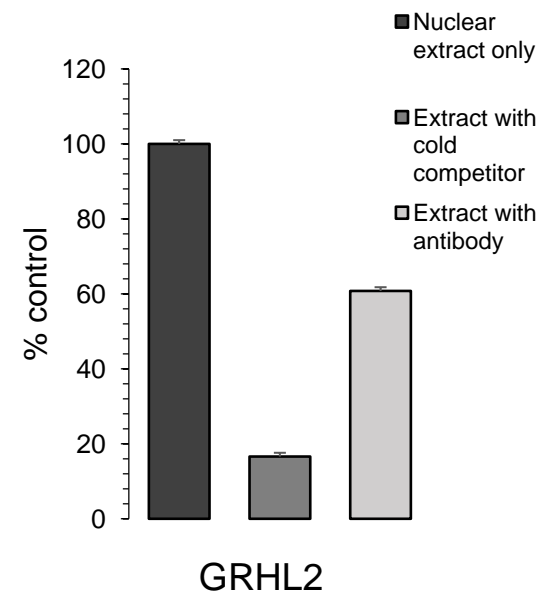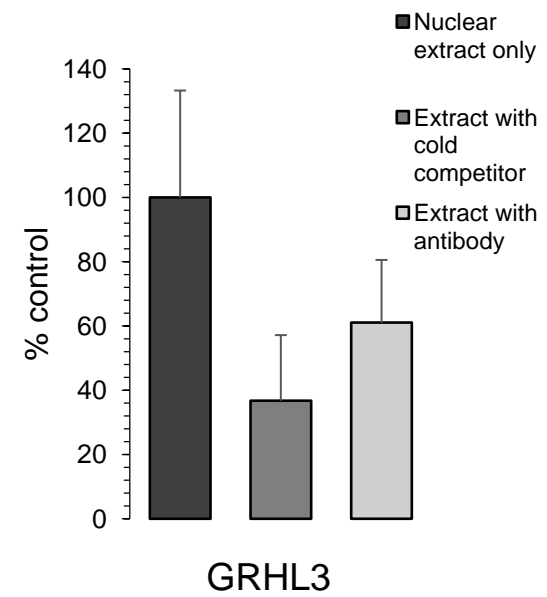**B**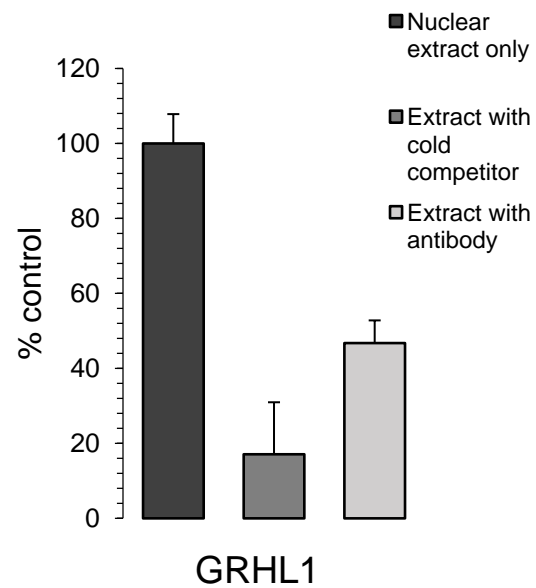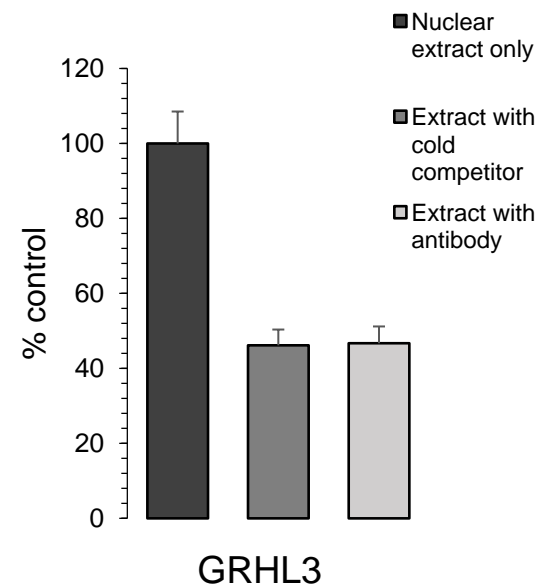

Supplement: S1 Fig — Quantitative results of EMSA experiments performed with probes including KLF4 (A) or PAX5 (B) binding sequences. The relative decrease in signals for cold probe or probe with KLF4 or PAX5 antibody compared with probes with nuclear extract (100%) was measured by densitometry (ImageJ). Data are shown as means ± SEM of two or three experiments. * one experiment. (PDF) [file pone.0257977.s001.pdf]
