# Supplementary material for: Transcription factors Krüppel-like factor 4 and paired box 5 regulate the expression of the Grainyhead-like genes
Source: PLoS One. 2021 Sep 27;16(9):e0257977. doi: 10.1371/journal.pone.0257977 (PMC8476022; doi:10.1371/journal.pone.0257977)
Supplement: S1 Table — (DOC) [file pone.0257977.s002.doc]

| **Vector name** | **Direction** | **Sequences of oligonucleotides (5'→3')** |
| --- | --- | --- |
| KLF4/GRHL1-luc | F | TATCGATAGGTACCGAGGAGGCGGCTGTACGGCCGGGGCTCGTCGCGAAGGGGATGGCCGGGGAC |
|  | R | GATCGCAGATCTCGAGTCCCCGGCCATCCCCTTCGCGACGACCCCCGGCCGTACAGCCGCCTCCT |
| NoKLF4/GRHL1-luc | F | TATCGATAGGTACCGAGGAGGCGGCTGTACGGCCGCGAAGGGGATGGCCGGGGAC |
|  | R | GATCGCAGATCTCGAGTCCCCGGCCATCCCCTTCGCGGCCGTACAGCCGCCTCCT |
| KLF4/GRHL2-luc | F | TCTTACGCGTGCTAGTCCTCACTCCATCAAAACCACGCCACTCCCCACCTGCCCAGCTCGTAAAAGGATGCTCAC |
|  | R | GATCGCAGATCTCGAGTGAGCATCCTTTTACGAGCTGGGCAGGTGGGGAGTGGCGTGGTTTTGATGGAGTGAGGA |
| NoKLF4/GRHL2-luc | F | TCTTACGCGTGCTAGTCCTCACTCCATCAAAACCACACCTGCCCAGCTCGTAAAAGGATGCTCAC |
|  | R | GATCGCAGATCTCGAGTGAGCATCCTTTTACGAGCTGGGCAGGTGTGGTTTTGATGGAGTGAGGA |
| KLF4/GRHL3-luc | F | TCTTACGCGTGCTAGGTACCCCCTCTCCAGAGCCCTGACCCCACCCCAAGAGGGCTCCTTTGAATTCTGACTTTA |
|  | R | GATCGCAGATCTCGATAAAGTCAGAATTCAAAGGAGCCCTCTTGGGGTGGGGTCAGGGCTCTGGAGAGGGGGTAC |
| NoKLF4/GRHL3-luc | F | TCTTACGCGTGCTAGGTACCCCCTCTCCAGAGCCCTGAAGAGGGCTCCTTTGAATTCTGACTTTA |
|  | R | GATCGCAGATCTCGATAAAGTCAGAATTCAAAGGAGCCCTCTTCAGGGCTCTGGAGAGGGGGTAC |
| PAX5/GRHL1-luc | F | TATCGATAGGTACCGCCCGCGCCGCTCGTCCCCGTCAGCCCCGCCCCGGCGCTCTTCTCGCCGACCAATCAGGAG |
|  | R | GATCGCAGATCTCGACTCCTGATTGGTCGGCGAGAAGAGCGCCGGGGCGGGGCTGACGGGGACGAGCGGCGCGGG |
| NoPAX5/GRHL1-luc | F | TATCGATAGGTACCGCCCGCGCCGCTCGTCCCCGTTCTCGCCGACCAATCAGGAG |
|  | R | GATCGCAGATCTCGACTCCTGATTGGTCGGCGAGAACGGGGACGAGCGGCGCGGG |
| PAX5/GRHL3-luc | F | TATCGATAGGTACCGAGAGCTCCCACTGACCCACCGGAGGAGTGAAGAGGGAAAACGGGGCTGAAACCCAGATGG |
|  | R | GATCGCAGATCTCGACCATCTGGGTTTCAGCCCCGTTTTCCCTCTTCACTCCTCCGGTGGGTCAGTGGGAGCTCT |
| NoPAX5/GRHL3-luc | F | TATCGATAGGTACCGAGAGCTCCCACTGACCCACCCGGGGCTGAAACCCAGATGG |
|  | R | GATCGCAGATCTCGACCATCTGGGTTTCAGCCCCGGGTGGGTCAGTGGGAGCTCT |
| KLF4/GRHL1-luc2 | F | TATCGATAGGTACCGAGGAGGCGGCTGTACGGCCGGGGCTCGTCGCGAAGGGGATGGCCGGGGAC |
|  | R | GATCGCAGATCTCGAGTCCCCGGCCATCCCCTTCGCGACGACCCCCGGCCGTACAGCCGCCTCCT |
| KLF4/GRHL1-SNP-luc2 | F | TATCGATAGGTACCGAGGAGGCGGCTGTACGGCCGGGG**T**TCGTCGCGAAGGGGATGGCCGGGGAC |
|  | R | GATCGCAGATCTCGAGTCCCCGGCCATCCCCTTCGCGACGA**A**CCCCGGCCGTACAGCCGCCTCCT |
|  | R | GATCGCAGATCTCGATCAGCGCTCCATAAATGTTGGC |

**S1 Table.** **List of oligonucleotides used for cloning.**
