# Supplementary material for: Transcription factors Krüppel-like factor 4 and paired box 5 regulate the expression of the Grainyhead-like genes
Source: PLoS One. 2021 Sep 27;16(9):e0257977. doi: 10.1371/journal.pone.0257977 (PMC8476022; doi:10.1371/journal.pone.0257977)
Supplement: S2 Table — (DOC) [file pone.0257977.s003.doc]

| **Binding site** | **Primer** | **Sequence (5'→3')** |
| --- | --- | --- |
| KLF4 in *GRHL1* promoter | F | ACAGCCTGCGAGAACCGAGA |
|  | R | CCGCTCATTGGCTGGCGAT |
| KLF4 in *GRHL2* promoter | F | CAGGCTCTGAACCACTAGGC |
|  | R | GTCTGCCTCCTGCCACTATC |
| KLF4 in *GRHL3* promoter | F | TCAAACCCAGTACCCCCTCT |
|  | R | GGCCCACTCCTCTCTCTCTT |
| PAX5 in *GRHL1* promoter | F | ATTGGTCGGCGAGAAGAGC |
|  | R | TCCCTCTCCTGTCAGGGC |
| PAX5 in *GRHL3* enhancer | F | GAGACAAGCGGTCAGCAG |
|  | R | GCAGGTATCCACCGATGTTTAC |
| ZNF333 | F | TGCAGCCAGTGTGGGAAAGC |
|  | R | GTGCTCGTCCGGAAGGGCTTG |
| KRAS | F | gcctgctgaaaatgactg |
|  | R | ggtcctgcaccagtaatatg |

**S2 Table.** **List of ChIP qRT-PCR primers.**
