# Supplementary material for: Transcription factors Krüppel-like factor 4 and paired box 5 regulate the expression of the Grainyhead-like genes
Source: PLoS One. 2021 Sep 27;16(9):e0257977. doi: 10.1371/journal.pone.0257977 (PMC8476022; doi:10.1371/journal.pone.0257977)
Supplement: S3 Table — (DOC) [file pone.0257977.s004.doc]

| **Binding site** | **Sequence (5'→3')** |
| --- | --- |
| KLF4 in *GRHL1* promoter | TACGGCCGGGGCTCGTCGCGAAG |
| KLF4 in *GRHL1* promoter with SNP variant | TACGGCCGGGG**T**TCGTCGCGAAG |
| KLF4 in *GRHL2* promoter | ACCACGCCACTCCCCACCTG |
| KLF4 in *GRHL3* promoter | TCTGGACCCCACCCCTCCCC |
| PAX5 in *GRHL1* promoter | CCCGTCAGCCCCGCCCCGGCGCTCTTCTCG |
| PAX5 in *GRHL3* enhancer | CCACCGGAGGAGTGAAGAGGGAAAACGGGG |

**S3 Table.** **List of EMSA oligonucleotide probes.**
