# Supplementary material for: Transcription factors Krüppel-like factor 4 and paired box 5 regulate the expression of the Grainyhead-like genes
Source: PLoS One. 2021 Sep 27;16(9):e0257977. doi: 10.1371/journal.pone.0257977 (PMC8476022; doi:10.1371/journal.pone.0257977)
Supplement: S4 Table — (DOC) [file pone.0257977.s005.doc]

| **Transcription factor** | **Binding sequence** | **P aposteriori** | **Weight matrix score** | **Position in analyzed regions** | **Strand** |
| --- | --- | --- | --- | --- | --- |
| KLF4 in *GRHL1* | GGGGCGGGGG | 0.648167 | 7.86877 | 1819-1828 | - |
| KLF4 in *GRHL1* | GGGGCGGGGA | 0.218157 | 7.7715 | 2523-2532 | - |
| KLF4 in *GRHL2* | GGGGAGTGGC | 0.829723 | 8.18977 | 1350-1359 | - |
| KLF4 in *GRHL2* | GGGGCTTGGC | 0.444678 | 5.3061 | 2799-2808 | + |
| KLF4 in *GRHL3* | AGGGCGGAGC | 0.998057 | 7.86071 | 1978-1987 | - |
| KLF4 in *GRHL3* | GGGGTGGGGT | 0.931358 | 9.17701 | 698-707 | - |
| KLF4 in *GRHL3* | GGGGTGGGGT | 0.603743 | 9.17701 | 962-971 | + |
| KLF4 in *GRHL3* | AGAGCGTGGA | 0.504012 | 2.3753 | 2281-2290 | + |
| PAX5 in *GRHL1* | AGAGCGCCGGGGCGGGGCTG | 0.993845 | 10.8085 | 1853-1872 | - |
| PAX5 in *GRHL1* | AAGGCTGGGAGGAAGAGCCA | 0.982394 | 8.6306 | 152-171 | - |
| PAX5 in *GRHL1* | AAGCAATGGAAGCGTGGAAG | 0.970851 | 6.03608 | 97-116 | - |
| PAX5 in *GRHL1* | AGGGGCTTGGAGCAGGGTAG | 0.892077 | 6.84752 | 682-701 | - |
| PAX5 in *GRHL1* | TGGCCGCGGAGGAGGGGAGG | 0.846382 | 9.65173 | 2410-2429 | - |
| PAX5 in *GRHL1* | GGACGAGCGGCGCGGGGCGG | 0.808400 | 9.13331 | 1829-1848 | - |
| PAX5 in *GRHL1* | CGCTCGCCGCTGCCGGGCCG | 0.774720 | 5.48255 | 2116-2135 | - |
| PAX5 in *GRHL1* | GAGGCCTCGGGGCTGGGAGG | 0.768389 | 7.59371 | 1903-1922 | - |
| PAX5 in *GRHL1* | AGGGCACAGAGGAGGGACTG | 0.753453 | 6.72677 | 18-37 | + |
| PAX5 in *GRHL1* | GGGTGGCTGGTGGGGCACAG | 0.677451 | 5.17656 | 816-835 | - |
| PAX5 in *GRHL1* | GAGGCTGCGGGGCACAGACA | 0.668394 | 5.2443 | 1261-1280 | + |
| PAX5 in *GRHL1* | GGGGCCGCGCTGAGGGGCCG | 0.634609 | 11.5418 | 2195-2214 | + |
| PAX5 in *GRHL1* | AGGAGAGGGGGGCGGGGAGG | 0.569628 | 9.49677 | 2521-2540 | - |
| PAX5 in *GRHL1* | GGGCCGTCGCGGTGGAGTGG | 0.510806 | 5.60408 | 1169-1188 | + |
| PAX5 in *GRHL1* | TGGGCCAGGAGAAGGGACAT | 0.495928 | 3.75782 | 1360-1379 | + |
| PAX5 in *GRHL1* | AGGCTCCCGAGGCCGACCGG | 0.460127 | 3.91608 | 2543-2562 | - |
| PAX5 in *GRHL1* | GGGCCACGAAGGTGGGACTG | 0.457174 | 4.92714 | 884-903 | - |
| PAX5 in *GRHL1* | GGCTCTCGGGGGAAGCCACG | 0.456123 | 4.21964 | 1389-1408 | + |
| PAX5 in *GRHL1* | GAAGACCTGGGGCATAGAAG | 0.411535 | 7.02001 | 403-422 | - |
| PAX5 in *GRHL1* | AGGGCAATAGAGTGAGGTGG | 0.395662 | 4.13012 | 579-598 | - |
| PAX5 in *GRHL1* | GGGCCGCCGCTCCGGACCCG | 0.279464 | 3.32072 | 2030-2049 | + |
| PAX5 in *GRHL1* | CGGCTCGGGTCCGGAGCGG | 0.244066 | 3.51957 | 2036-2055 | - |
| PAX5 in *GRHL1* | CGGCCGCCGCGGGGGGGCCG | 0.242143 | 8.66426 | 2182-2201 | + |
| PAX5 in *GRHL1* | TGCTGCGTGGTGGGGACCAG | 0.218395 | 3.45611 | 628-647 | - |
| PAX5 in *GRHL1* | CGGAGCCTCGGGAGGAGAGA | 0.204767 | 3.51811 | 2350-2369 | + |
| PAX5 in *GRHL3* | GGAGGAGTGAAGAGGGAAAA | 0.980442 | 8.9417 | 2386-2405 | + |
| PAX5 in *GRHL3* | GGAGCTTGGGAGCAGGAAGA | 0.722296 | 8.33589 | 2212-2231 | + |
| PAX5 in *GRHL3* | GGGGGAGGGGAGAATAGAAA | 0.518086 | 6.62923 | 873-892 | + |
| PAX5 in *GRHL3* | AGGGCTCTGCAGAGAACAAA | 0.431951 | 4.06235 | 480-499 | + |

**S4 Table. List of identified putative binding sites for KLF4 and PAX5 in the regulatory regions of *GRHL1-3* genes.**
