# Supplementary material for: Transcription factors Krüppel-like factor 4 and paired box 5 regulate the expression of the Grainyhead-like genes
Source: PLoS One. 2021 Sep 27;16(9):e0257977. doi: 10.1371/journal.pone.0257977 (PMC8476022; doi:10.1371/journal.pone.0257977)
Supplement: S1 Raw images — (PDF) [file pone.0257977.s008.pdf]

Fig. 2B (GRHL1)

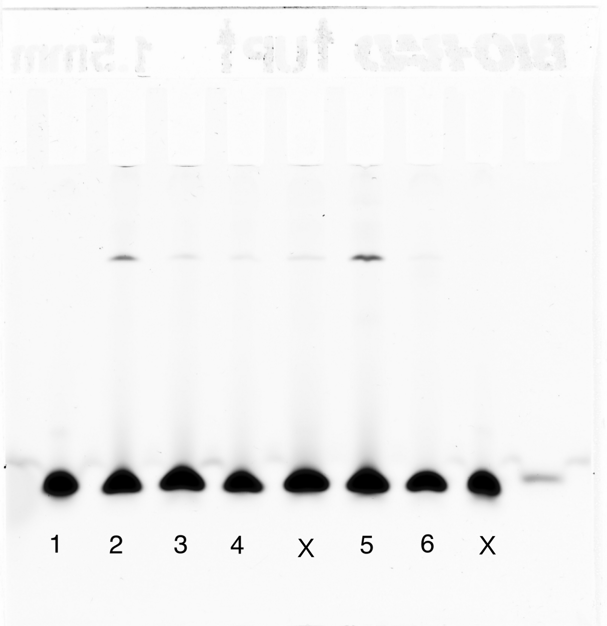

Loading order: from left to right.

Numbers: experimental samples Fig. 2B (GRHL1).

Image captured on Typhoon FLA 9000 laser scanner, fluorescence method.

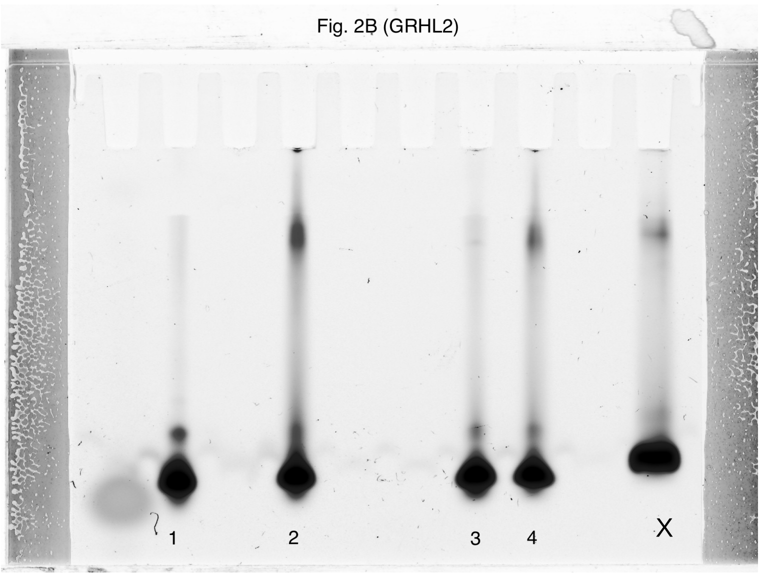

Loading order: from left to right.

Numbers: experimental samples: Fig. 2B(GRHL2).

Image captured on Typhoon FLA 9000 laser scanner, fluorescence method.

Fig. 2B (GRHL3)

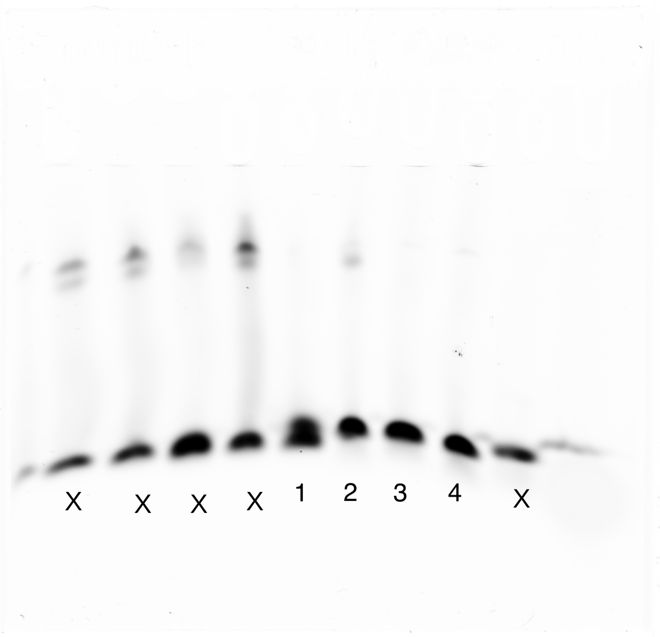

Loading order: from left to right.

Numbers: experimental samples: Fig. 2B(GRHL3).

Image captured on Typhoon FLA 9000 laser scanner, fluorescence method.

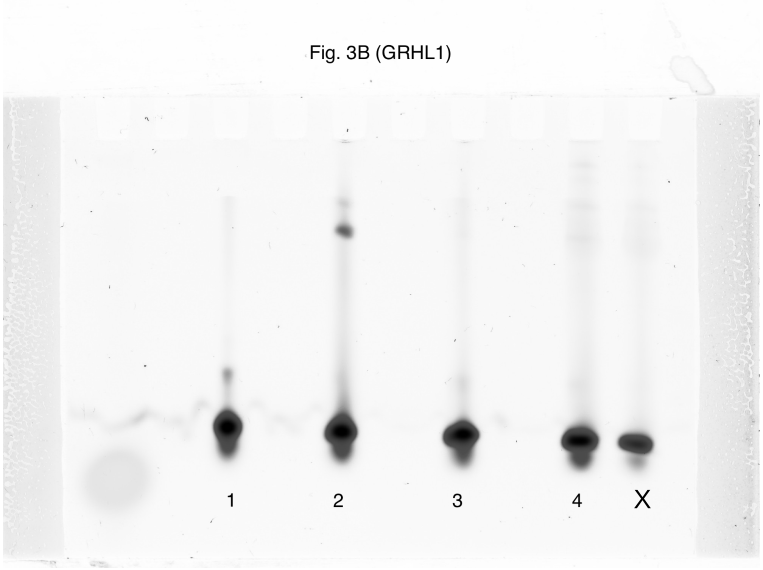

Loading order: from left to right.

Numbers: experimental samples: Fig. 3B(GRHL1).

Image captured on Typhoon FLA 9000 laser scanner, fluorescence method.

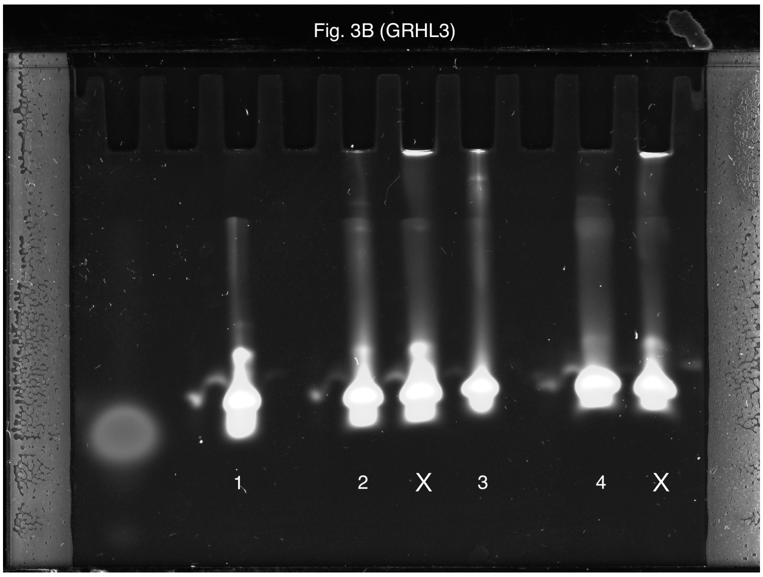

Loading order: from left to right.
Numbers: experimental samples: Fig. 3B(GRHL3).
Image captured on Typhoon FLA 9000 laser scanner, fluorescence method.
